# Infection with the enteric pathogen *C. rodentium* promotes islet-specific autoimmunity by activating a lymphatic route from the gut to pancreatic lymph node

**DOI:** 10.1038/s41385-022-00490-2

**Published:** 2022-02-09

**Authors:** Sakari Pöysti, Raine Toivonen, Akira Takeda, Satu Silojärvi, Emrah Yatkin, Masayuki Miyasaka, Arno Hänninen

**Affiliations:** 1 Institute of Biomedicine, University of Turku, Turku, Finland; 2 Department of Clinical Microbiology and Immunology, Turku University Hospital, Turku, Finland; 3 MediCity Research Laboratory, University of Turku, Turku, Finland; 4 Central Animal Laboratory, University of Turku, Turku, Finland; 5 Immunology Frontier Research Center, Osaka University, Suita, Japan; 6 InFLAMES Research Flagship Center, University of Turku, Turku, Finland

## Abstract

In nonobese diabetic (NOD) mice, *C. rodentium* promotes priming of islet-specific T-cells in pancreatic lymph nodes (PaLN), which is a critical step in initiation and perpetuation of islet-autoimmunity. To investigate mechanisms by which *C. rodentium* promotes T-cell priming in PaLN, we used fluorescent imaging of lymphatic vasculature emanating from colon, followed dendritic cell (DC) migration from colon using photoconvertible-reporter mice, and evaluated the translocation of bacteria to lymph nodes with GFP-*C. rodentium* and in situ hybridization of bacterial DNA. Fluorescent dextran injected in the colon wall accumulated under subcapsular sinus of PaLN indicating the existence of a lymphatic route from colon to PaLN. Infection with *C. rodentium* induced DC migration from colon to PaLN and bacterial DNA was detected in medullary sinus and inner cortex of PaLN. Following infection with GFP-*C. rodentium*, fluorescence appeared in macrophages and gut-derived (CD103+) and resident (CD103-/XCR1+) DC, indicating transportation of bacteria from colon to PaLN both by DC and by lymph itself. This induced proinflammatory cytokine transcripts, activation of DC and islet-specific T-cells in PaLN of NOD mice. Our findings demonstrate the existence of a direct, enteric pathogen-activated route for lymph, cells, and bacteria from colon, which promotes activation of islet-specific T-cells in PaLN.

## Introduction

Microbes in the gastrointestinal tract generate immune responses which are mostly physiological and promote immune tolerance in the healthy gut^1,2^. Such immune responses impact not only on the gut itself but also on regulation of metabolism and self-tolerance in the whole organism^3^. Lack of species diversity, colonization of the gut with microbes not adapted to a symbiotic lifestyle, or overgrowth of microbial species capable of outcompeting useful symbionts leads to disturbances in the interplay between microbes and gut immune system, referred to as dysbacteriosis or dysbiosis^4,5^. Dysbiosis links clearly to the pathogenesis of inflammatory bowel disease^6,7^, but it may also be involved in the pathogenesis of common autoimmune diseases^8,9^ including type 1 diabetes (T1D)^10,11^. Dysbiosis predisposes to cellular stress in villous epithelium, compromises epithelial barrier function and allows luminal microbes to access layers where they may ignite inflammatory immune responses^12^, but the mechanisms by which dysbiosis reflects to T1D are still mostly speculative^13^.

Compromised epithelial barrier function increases gut permeability, which is increased in many individuals prior to T1D onset^14^ and appears to have a role in the pathogenesis of T1D^15^. In animal models of T1D, aggravating gut permeability by dextran sodium sulphate (DSS)^16^ and infection with the enteric pathogen *Citrobacter rodentium* enhances activation of islet-specific T cells in pancreatic lymph nodes^17^ and accelerates T1D development (unpublished data). In the low-dose streptozotocin model of T1D autoimmunity, perturbation of gut microbiota associated with an increase in bacterial 16S-RNA-encoding DNA^18^ and enhanced differentiation of Th1 and Th17 helper T cells in pancreatic lymph nodes. Recently, distinct lymph nodes dedicated to collecting lymph and migratory DC from the colon were identified^19,20^, and their role in gut immunity has thereafter been studied using powerful model systems^21^. They are located in the mesenterium of the large intestine close to the pancreas^20^, but pancreatic lymph nodes are distinct from these lymph nodes^22–24^.

To investigate mechanisms by which dysbiosis may enhance priming T cells in pancreatic lymph nodes, we focused on the possibility of a lymphatic connection between the colon and pancreatic lymph nodes. We injected fluorescent dye into the serosa of small intestine and colon, and followed its dissemination via lymphatics by microscopic imaging of colon-draining and pancreatic lymph nodes. We also tracked dendritic cell migration from colon to these lymph nodes using mice with constitutive expression of a photoconvertible reporter gene, and imaged microbial DNA in lymph nodes of NOD mice using 16S-RNA in situ hybridization. To investigate the mechanisms by which enteric bacteria are transported to pancreatic lymph nodes, we infected mice with *C. rodentium* transfected with the fluorescent protein GFP and followed its appearance in colon-draining and pancreatic lymph nodes and in myeloid cells in these organs. Our results are in evidence of a lymphatic route from colon to pancreatic lymph node, which allows migration of cells and dissemination of microbes following infection with the enteric pathogen *C. rodentium*. This novel lymphatic route may constitute a physical link between gut dysbiosis and islet-autoimmunity.

## Results

### Lymphatics from proximal colon reach pancreatic lymph nodes

**Fig. 1:**
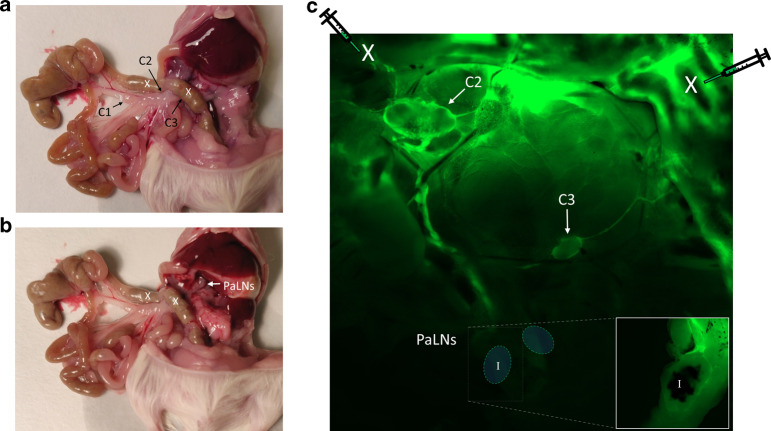
FITC-dextran injected in colon wall accumulates in colon-draining and in pancreatic lymph nodes. **a** A photograph of a dissected mouse showing colon-draining (coMLN) lymph nodes C1-C3 located in the mesenterium; and **b** pancreatic (PaLN) lymph nodes located in the rear of peritoneal cavity. **c** A photomicrograph showing diffusion of FITC dextran injected into colon wall (injection sites marked by an X). FITC-dextran accumulates under subcapsular sinus of two colon-draining lymph nodes (C2, C3, arrows) and under subcapsular sinus of a separate lymph node located at the rear of peritoneal cavity (PaLN). Due to their location close to vertebral column, PaLN were visible only after further preparation (inset). During dissection, PaLN block was marked with black ink (I in the insert) to help its identification.

Several studies have linked mild colonic inflammation or a change in colon microbiota composition to increased activation of islet-specific T cells or increased Th1 and Th17 differentiation of T cells in pancreatic lymph node (PaLN)^16–18^. To address the possibility that a lymphatic route between colon and PaLN facilitates this, we injected small amounts of fluorescent dextran in colon wall as described previously^20^ and allowed it to diffuse along with lymph to local lymph nodes (Fig. 1a, b). Injections near caecum were only able to load first coMLN (C1)^21^ and no visible FITC-DX accumulated around PaLN. After injecting FITC-Dx into two separate spots in the wall of proximal colon and distal colon of anesthetized NOD mice, fluorescent dextran accumulated in colon-draining lymph nodes known as C2 and C3^21^, but also in PaLN (Fig. 1c). Due to its location in the rear of peritoneal cavity, lymphatic vasculature emanating from the intestine became obscured before reaching PaLN, but accumulation of fluorescence could be clearly visualized under the capsule of PaLN (Fig. 1c insert). To ensure it is identical to the lymph node which is the site of proliferation of islet-specific T cells (see below) it was marked with black ink before dissection of the tissue block (Fig. 1a, b). Accumulation of fluorescent dextran under PaLN capsule took place only after its injection into colon wall, since no detectable FITC signal was found after injections into small intestine (Supplementary Fig. 1).

### Soluble antigen originating from colon wall is captured by macrophages and DC in pancreatic lymph nodes

**Fig. 2:**
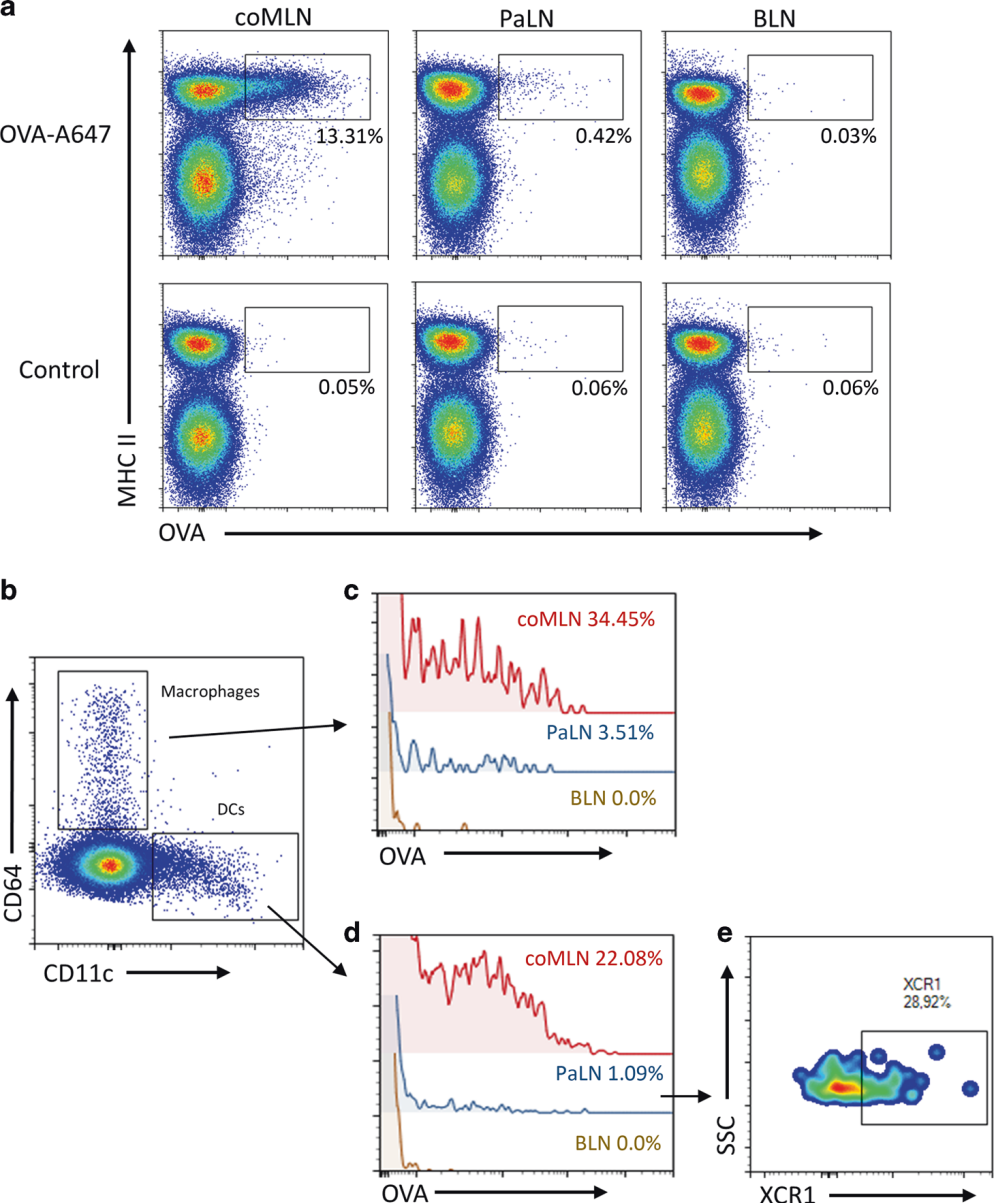
Soluble antigen injected subserosally in colon wall is endocytosed by macrophages and DC in coMLN and PaLN. **a** Injection of OVA-A647 (upper panels) leads to a fluorescent signal in MHCII+ cells in coMLN and PaLN but not in BLN (brachial lymph node) as compared to PBS injection (“control”, lower panels). **b** MHCII+ cells divided into macrophages (MHCII^+^/CD64^+^) and DCs (MHCII^+^/CD11c^+^). **c** OVA-A647 positivity in CD64^+^MHCII^+^ macrophages in coMLN and PaLN. **d** Also CD11c^+^MHCII^+^ DC show OVA-A647 positivity in coMLN and PaLN. **e** XCR1 expression in OVA-A647 positive DCs. Lymph nodes were analyzed 1 h after injection of OVA-A647. In (**c** and **d**) background was reduced from the frequencies of positive cells indicated in histograms. Single live cells were gated as CD45^+^/CD19^−^/TCRβ^−^ cells before further population identification. In figure **a** percentages represents portions from MHCII+ cells. Regions were gated according to FMO stainings. Data are representative of 4 mice/group and two independent experiments.

As antigens in afferent lymph are taken up by myeloid cells in draining lymph nodes, we evaluated if OVA-A647 is taken up by macrophages and DC in coMLN and PaLN following its injection subserosally in colon wall (Fig. 2). In case a lymphatic connection exists between colon and PaLN, we surmised that OVA-A647 is taken up also in PaLN. We analyzed MHC II^+^ cells for positivity to OVA-A647 fluorescence briefly (50–60 min) after injecting it directly to colon wall. OVA-A647 was taken up by MHCII^+^ cells in coMLN and PaLN but not in brachial lymph nodes (BLN) (Fig. 2a). Further analysis of the MHCII^+^ cell populations showed that CD64^+^ macrophages uptake OVA in the coMLN and PaLN (Fig. 2b, c). OVA-A647 positivity was also seen in MHCII^+^/CD11c^+^ dendritic cells (Fig. 2d) but in this short course of the experiment, not to the same extent as in CD64+ macrophages. No significant OVA signal was seen in CD11c^−^/CD64^−^ cells (data not shown). To evaluate if OVA-A647 positive DC has capabilities of antigen cross-presentation^25^, we analyzed XCR1 expression (Fig. 2e) and detected that a proportion of OVA-A647 positive DC express XCR1. From OVA positive XCR1^-^ DCs 63% of the cells were CD11b^+^/CD103^−^ cells (data not shown).

### Infection with *C. rodentium* induces migration of DC from colon to pancreatic lymph nodes

**Fig. 3:**
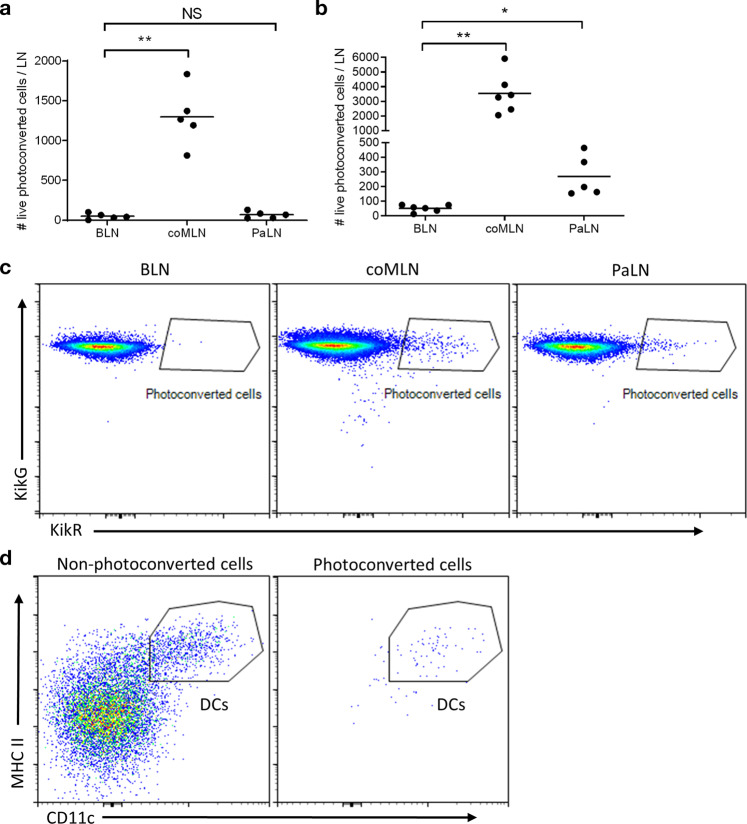
DC migrate from colon to PaLN only during dysbiosis. **a** Under steady-state conditions, photoconverted cells are found in coMLNs but not in PaLN or BLN. (**b**, **c**) Following photoconversion of colon wall after *C. rodentium* infection, photoconverted (KikRHI) cells are found also in PaLN. **b** Phenotyping of photoconverted cells in PaLN identifies them as MHCII^HI^ CD11c^HI^ DC. **p ≤ 0.01, ***p ≤ 0.001. Single live cells were gated as CD45^+^/CD19^−^/TCRβ^−^ before further population identification. Data are from four independent experiments and each data point represents one mouse. One-way ANOVA and Dunnett’s T3 multiple comparisons test was used to compare groups.

To explore if dendritic cells migrate from colon to PaLN we used mice constitutively expressing the photoconvertible Kikume-protein^26^. Consistent with earlier studies^19^, we observed photoconverted cells in coMLN (Fig. 3a, b) following exposure of the surface of proximal colon to UV light. However, no photoconverted cells were observed in PaLN or in small intestinal (siMLN) lymph nodes.

To analyze migration of colon DC during perturbation of microbial homeostasis, mice were infected with *C. rodentium*. In healthy B6 mice including our local colony, *C. rodentium* infection presents with relatively mild clinical symptoms despite of inflammatory changes in the gut^27,28^. Microbiota profiling revealed that *Citrobacter* infection led to colonization by the pathogen itself, but also altered the abundances of several bacterial taxa (Supplementary Fig. 2), some of which may be of relevance for autoimmunity in NOD mice^29^. *C. rodentium* infection increased the number of photoconverted cells in the coMLN, and in addition, photoconverted cells became identifiable also in PaLN (Fig. 3b, c). Phenotypic analysis identified these as CD11c^+^/MHCII^HI^ dendritic cells (Fig. 3d).

### Bacterial DNA in pancreatic lymph nodes is increased by *C. rodentium*

**Fig. 4:**
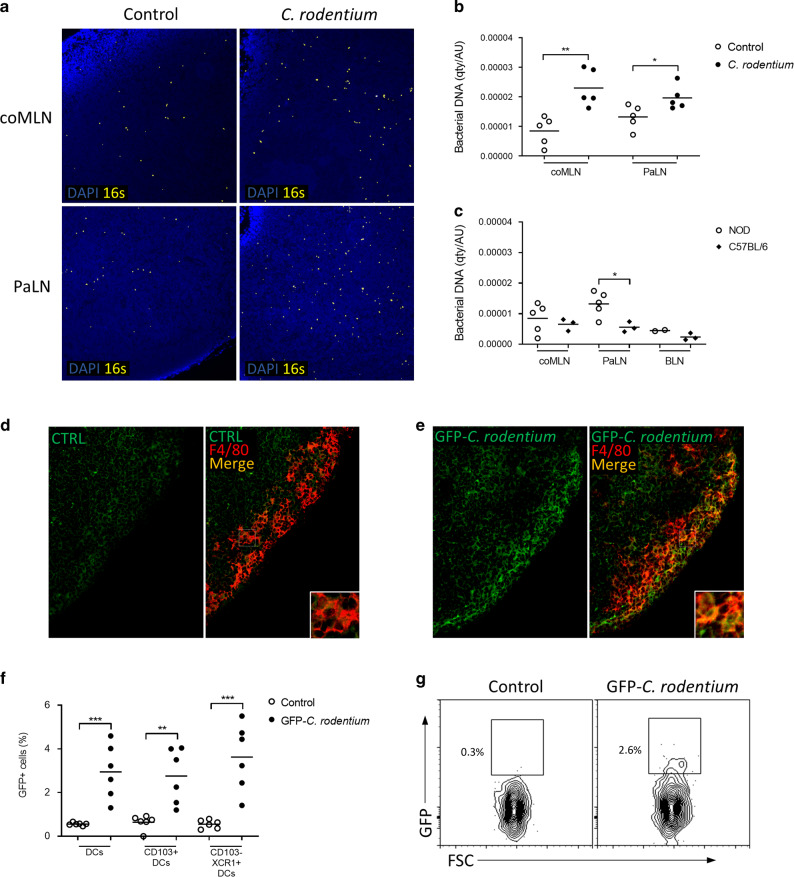
Dysbiosis increases translocation of bacterial DNA to coMLN and PaLN. **a** Photomicrographs of coMLN and PaLN sections (20x magnification) of NOD mice following in situ hybridization for bacterial 16S RNA gene. **b** Bacterial DNA is increased significantly in both coMLN and PaLN sections in NOD mice infected with *C. rodentium*. **c** NOD mice have significantly more bacterial DNA in PaLN also during steady state when compared to C57BL/6 mice. GFP signal in medullary sinus of PaLN after infecting mice with GFP-*C. rodentium* (**e**) compared to control mice (**d**). GFP is co-localized with F4/80^+^ cells (20x magnification). **f** Increased GFP positivity in DCs (MHCII^+^/CD11c^+^ cells), and in CD103^+^ DCs and CD103^−^/XCR1^+^ DCs after oral inoculation of GFP-*C.rodentium*. **g** Representative flow cytometry dot plots of GFP signal in MHCII^+^/CD11c^+^ DCs. For flow cytometry, live single cells were gated as CD45^+^/CD19^−^/TCRβ^−^ before further population identification. *p ≤ 0.05, **p ≤ 0.01 (unpaired Student’s *t* test). Bacterial counts (**b, c**) were quantified using ImageJ software and are expressed as qty/AU (quantity/arbitrary units). Data in each panel are from three independent experiments. Each data point represents an individual mouse.

In situ hybridization for 16S-RNA gene allows localization of bacterial DNA in tissue sections. Seven days after *C. rodentium* infection, bacterial DNA-derived fluorescence was more widely dispersed and more intense in both coMLN and PaLN (Fig. 4a, b) compared to non-infected mice. Irrespective of *C. rodentium* treatment, Bacterial DNA was accentuated in inner cortex, while rounded areas in outer cortex suggestive of B-cell follicles were mostly devoid of bacterial DNA. Even without *C. rodentium* administration, bacterial DNA was detected in PaLN of NOD mice at higher levels than in C57BL/6 mice (Fig. 4c) consistent with inherent low-level dysbiosis in NOD mice^29^.

To study in more detail the translocation of colon-originated bacteria to PaLN, we infected the mice with GFP-expressing *C. rodentium*. Immunostaining of the lymph node sections showed accumulation of fluorescent signal especially in medullary sinus of the PaLN (Fig. 4d, e). GFP-derived fluorescence was identified to co-localize with F4/80^+^ macrophages (Fig. 4d, e). This suggests that GFP-*C. rodentium*, or parts of the bacteria reach the PaLN in soluble form transported by lymph itself, and is taken up there by macrophages. Analysis of CD11c^+^/MHCII^+^ dendritic cells 6 days after oral inoculation of GFP-*C. rodentium* (Fig. 4f, g) showed GFP signal both in CD103^+^ DC and in CD103^−^/XCR1^+^ DC which are supposedly capable of antigen cross-presentation^25^.

### *Citrobacter* infection increases cytokine expression and associates with activation of DC and T cells in PaLN

**Fig. 5:**
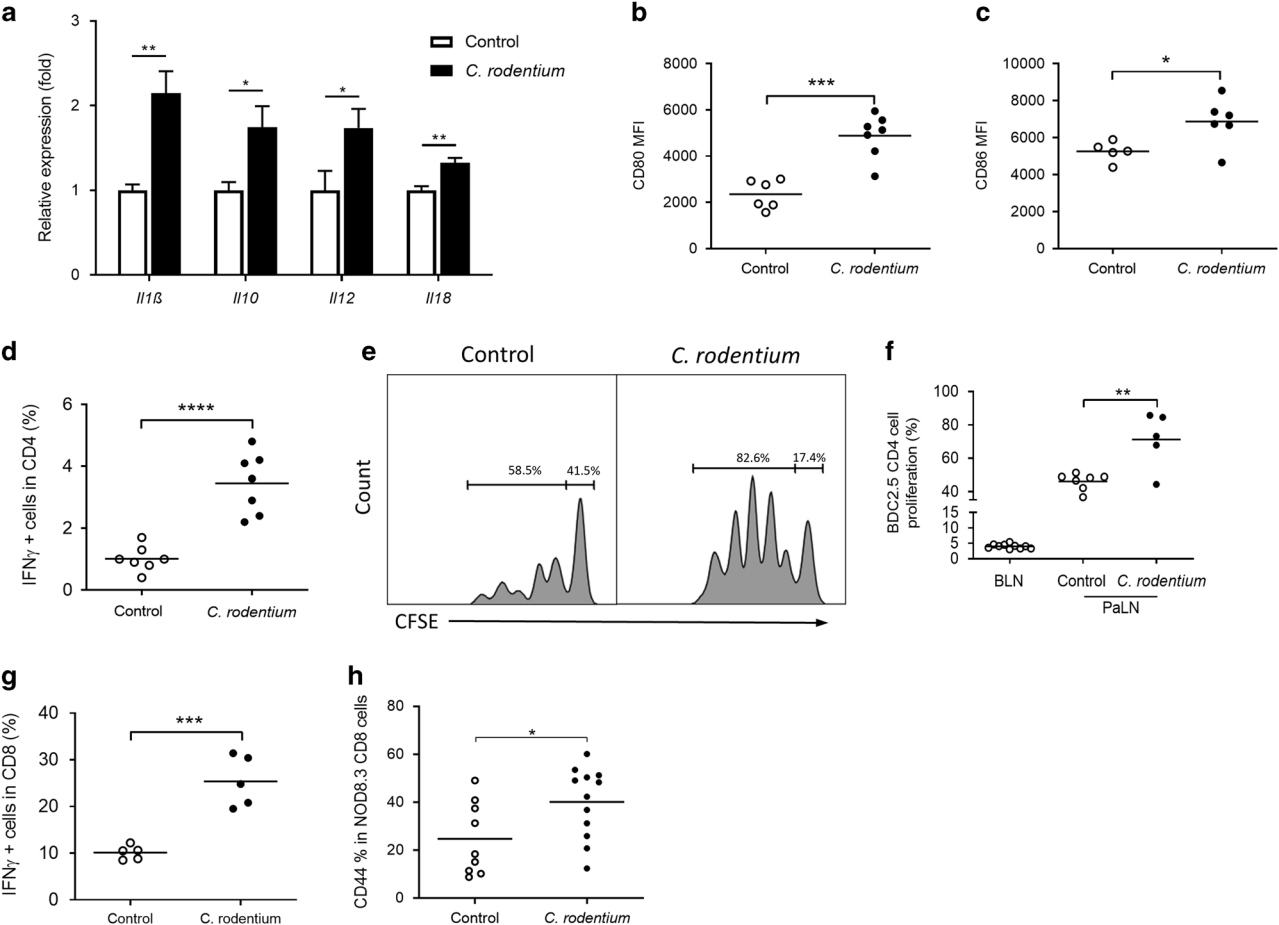
*C. rodentium* infection promotes DC activation and T cell response in PaLN of NOD mice. **a** Relative expressions of cytokine transcripts in PaLN of control and *C. rodentium* -infected mice (*n* = 6, data represent mean + SD). (**b**, **c**) CD80 and CD86 levels on CD11c^+^MHC II^+^ DC. **d** IFNγ production in CD4 T cells in PaLN. **e** Representative dilution of CFSE label among successive cell divisions in adoptively transferred islet-specific BDC2.5 T cells in PaLN of uninfected and *C. rodentium* infected mice. **f** *C. rodentium* infection increases BDC2.5 cell proliferation in PaLN. **g** IFNγ production in CD8 T cells in PaLN. **h** CD44 in IGRP-specific T cells in PaLN of 5 week old NOD.8.3 mice 6 days after *C. rodentium* infection. BLN, brachial lymph node. *p < 0.05; **p < 0.01; ***p < 0.001; ****p < 0.0001 (unpaired Student’s *t-test*). T cells were gated as single live cells and CD45^+^/CD19^−^/TCRβ^+^ before further population identification. Data are from 3 independent experiments. Each data point represents an individual mouse.

Following infection with *Citrobacter* 7 days earlier, the expression of transcripts for proinflammatory cytokines *Il1β*, *Il12*, and *Il18* and for immunoregulatory *Il10* was increased (Fig. 5a). Suggesting increased activation and co-stimulatory capacity of dendritic cells, CD80 and CD86 were expressed at significantly higher levels on CD11c^+^/MHCII^HI^ DC (Fig. 5b, c). These changes in PaLN coincided with increased percentage of IFNγ–expressing CD4 cells (Fig. 5d). Since *C. rodentium* accelerates diabetes development in NOD mice (unpublished data), we wanted to evaluate activation of islet-reactive T cells by adoptive transfer system of BDC2.5 T cells. BDC2.5 T cells bear a T-cell receptor originally derived from a diabetogenic T-cell clone in NOD mice^30^, and proliferate and expand in PaLN when adoptively transferred to NOD mice^31,32^. The fraction of BDC2.5 cells proliferating and undergoing cell division in PaLN was significantly enhanced in recipients of BDC2.5 T cells pre-treated with *C. rodentium* (Fig. 5e, f). When analyzing CD8 cells, we saw an increase also in IFNγ -producing CD8 cells after *C. rodentium* treatment (Fig. 5g). In 4 weeks old transgenic NOD.8.3 mice in which most CD8 T cells bear a T-cell receptor recognizing an islet-antigen, CD44 expression increased in their CD8 cells following *C. rodentium* infection (Fig. 5h).

## Discussion

Islet-autoimmunity initiates and is further promoted by the activation of islet-reactive T cells in pancreatic lymph node PaLN^32,33^. Microbial signals condition antigen-presenting cells to drive inflammatory and autoimmune Th1 and Th17 T-cell responses^2,34^, and among gut-draining lymph nodes, those draining distal parts of the intestine with high microbe content harbor dendritic cells driving mostly immune responses involving these subsets^21^. In nonobese diabetic (NOD) mice, activation of islet-reactive T cells in PaLN can be enhanced by inducing inflammation in the large intestine either by the chemical irritant DSS or by *C. rodentium* infection^16,17^. In the multiple low-dose streptozotocin model also involving microbiota perturbation in the large intestine, activation of Th1 and Th17 subsets is enhanced in PaLN via the microbial sensor NOD2^18^. In these studies, the presence of bacteria in PaLN was identified by quantitative PCR and in vitro culture methods^17,18^. To corroborate these findings and to investigate the route by which bacteria reach PaLN following perturbation of gut homeostasis, we imaged lymphatic vasculature from colon to draining lymph nodes, used in situ hybridization of bacterial DNA, and infected mice with *C. rodentium* to study the anatomic localization of microbes or their components in the lymph node topology and their association with DC subsets and macrophages present in PaLN. Following direct injections of fluorescent dextran into colon wall, we showed its absorption in lymphatic vasculature emanating from colon and its accumulation in PaLN, and following direct injections of fluorescent ovalbumin into colon walls, we observed its uptake in DC and macrophages in PaLN. We found that perturbing gut homeostasis with *C. rodentium* infection, allowed Kikume-red dendritic cells to migrate from colon not only to colon-draining^19,20^ but also to pancreatic lymph nodes. Concomitantly with this, bacteria emerged in medullary sinus of pancreatic lymph nodes and associated with macrophages and DC, which were able to present orally administered ovalbumin under conditions of increased gut permeability.

Dysbiosis impairs epithelial barrier, and allows dissemination of alarm-signals beyond gut environment^35^. Leaky gut appears to accompany the development of T1D in humans and in mouse^14,36,37^, and this may involve both systemic and compartmentalized mechanisms. Dysbiosis increases levels of bacterial endotoxin in circulating blood^38^ and gives rise to metabolic endotoxemia^39^ possibly conditioning DC and other immune cells via innate immune mechanisms in a generalized manner. Prior characterization of lymphatic vasculature draining lymph and DC from large intestine^19–21^ allowed to address if mechanisms related to colon immune surveillance are subject to changes during states of disturbed microbial homeostasis in the colon. While colon-draining mesenteric lymph nodes were previously identified as lymph nodes to which DC migrate from colon wall^19–21^, accumulation of fluorescent dextran under the capsule of pancreatic lymph node and of fluorescent ovalbumin in phagocytic cells in pancreatic lymph node after their injection to colon wall suggested that a lymphatic connection exists also between colon and pancreatic lymph node. We thus hypothesized that the physiologic compartmentalization of colon-draining lymph could be subject to de-regulation by factors disturbing physiologic homeostasis in the colon. Accordingly, *C. rodentium*-related inflammation and dysbiosis enabled DC and bacteria to migrate from colon to PaLN, suggesting that dysbiosis perturbs this compartmentalization and activates a route for lymph and lymph-borne cells and bacteria from colon to pancreatic lymph nodes.

According to earlier studies, activation of islet-specific T cells in vivo takes place in lymph nodes referred to as pancreatic lymph nodes (PLN/PaLN)^22–24^. Recently, 3-D imaging of lymphatic vessels and lymph nodes along the whole intestine documented the existence of lymph nodes denoted as pancreatic–duodenal lymph nodes, and draining the duodenum and separately the ascending and transverse colon^21^. This suggests that lymphatic drainage from the gut and the pancreas could intersect at one or several sites. It is also plausible that the omentum participates in the immune surveillance of the peritoneum and visceral organs, and thus acts as a gatekeeper of microbial insults^40^. Omentum contains unique immune structures called “milky spots” and lines visceral organs including stomach, intestine, liver and pancreas. Thus, omentum could play hitherto unrecognized roles in transporting antigens from the gut to peritoneal cavity or from peritoneal cavity to visceral organs, especially in circumstances of severe barrier disruption.

Under steady-state conditions, most DC in lymph nodes are resident DC^41^. Of these, those expressing the chemokine-receptor XCR1 associate with the capability to cross-present tissue-derived antigens^25^ and thus, XCR1 expression in a proportion of DC processing OVA-A647 in pancreatic lymph node suggested that some of the DC in pancreatic lymph node, which take up lymph-borne factors derived from colon, are also capable of antigen cross-presentation. This may render them subject to conditioning by microbial factors^42^. Within lymph nodes, cross-presentation enables processing of soluble antigens to elicit not only CD4 but also CD8 T-cell responses^43–46^. Enhanced activation of islet-specific CD4 T cells following perturbation of colon homeostasis in earlier^16,17^ and also CD8 T cells in the present study suggests that resident DC cross-presenting tissue-derived antigens from pancreatic islets become more activated if microbial structures reach pancreatic lymph nodes. Thus, immune tolerance to islet antigens may be particular sensitive to colon dysbiosis^42^.

In earlier studies, the presence of bacteria in pancreatic lymph nodes was documented by means of quantitative PCR and in vitro culture^17,18^, but their localization in the lymph node nor association with DC was not addressed in more detail. Imaging of bacterial DNA in PaLN by in situ hybridization and infection of mice with fluorescent *C. rodentium* allowed us to parallel bacterial signal with existing structures and cells in PaLN. Imaging of bacterial DNA by in situ hybridization showed its accumulation in medullary sinus of the lymph node, and fluorescent signal derived from GFP-expressing *C. rodentium* co-localized with F4/80+ macrophages indicating that bacteria reach pancreatic lymph nodes in soluble form. Infection with GFP-expressing *C. rodentium* indicated uptake of fluorescent signal also in dendritic cells of both migratory (CD103^+^) and cross-presenting (XCR1^+^) phenotypes. As most DC in lymph nodes are resident DC^41^. Uptake of bacteria by resident DC, some of which are capable of cross-presenting antigens, impacts likely more to presentation of islet antigens than uptake of bacteria in gut-derived migratory DC.

Perturbations in gut homeostasis associate with disease onset and islet autoimmunity in T1D and its animal models^14,16,17,37,47^, but the mechanisms underlying these associations and the association of microbial dysbiosis with autoimmunity in T1D development have remained speculative. The present study provides novel insight in these mechanisms by showing evidence of a lymphatic route from proximal colon to pancreatic lymph node in mice. Modeled by infection with *Citrobacter*, this lymphatic route became active under conditions of intestinal dysbiosis. Various infections including viral infections^48^ cause dysbiosis. Thus, similar effects are likely to accompany other infections as well. In humans, the primary lymphatic drainage from ascending colon runs adjacent to the pancreas, and metastases of colon carcinoma may appear in lymph nodes close to the pancreas^49^. Thus, a route of lymph from colon to pancreatic lymph nodes activated under pathologic conditions may also exist in humans. If lymphatic drainage from the colon shares commonalities between rodents and humans, it may be increasingly relevant to target gut dysbiosis for the prevention of type 1 diabetes.

## Methods

### Mice

NOD/ShiLtJ, NOD.BDC2.5 (NOD.Cg-Tg(TcraBDC2.5, TcrbBDC2.5)1Doi/DoiJ) and NOD.8.3 (NOD.Cg-Tg(TcraTcrbNY8.3)1Pesa/DvsJ) mice were purchased from Jackson Laboratories and maintained in Central Animal Laboratory of University of Turku (UTUCAL). KikGR mice, which express a photo convertible reporter protein in all cells^26^, were launched at UTUCAL via embryonic transfer using embryos received from Osaka University. The animals maintained in individually ventilated cages (IVC) and were fed *ad libitum*.

### *Citrobacter rodentium* infection and microbiota sequencing

*Citrobacter rodentium* was grown and administered as described earlier^27^. Each mice was given 1 × 10^9^ bacteria orally in 200 µl.

### Visualization of lymphatics

To investigate how lymphatic system of the colon is connected to pancreas, we injected FITC-labeled dextran to the colon wall. NOD mice were anesthetized using 150 mg/kg ketamine (Ketalar 50 mg/ml, Pfizer) and 10 mg/kg xylazine (Rompun vet 20 mg/ml, Bayer) mixture i.p. and peritoneal membrane and colon were gently pulled out from a small incision. Fluorescein isothiocyanate-labeled dextran 70kD (Sigma-Aldrich, Germany) was injected into colon wall. Injections were made into proximal parts of the colon. The incision was closed and mice were kept anesthetized for 30 min before sacrifice. A tissue block including cecum, colon, mesenteric LNs, pancreatic LNs and pancreas was prepared. Pancreatic LNs were marked with black marker to ensure correct identification. The block was glued onto petri dish and submerged in cold PBS. FITC signal was analyzed under stereo microscope (Zeiss AxioZoom.V16, Carl Zeiss, Germany). After the lymphatics were imaged, pancreatic LNs were separated from other tissues to visualize FITC-Dx loading into subcapsular sinus of the pancreatic LNs.

### Antigen flow from gut to lymph nodes

To analyze antigen uptake by APCs in lymph nodes, Alexa Fluor 647-labeled ovalbumin (OVA-A647, 1 mg/ml, Life Technologies, USA) was injected into the colon wall as described above. LNs were collected 1 h later, and DC and macrophage populations were analyzed by flow cytometry as described below.

### DC trafficking from colon to draining lymph nodes

KikGR mice constitutively express the photoconvertible Kikume-protein ubiquitously in all cells^26^. A 20 mm skin incision was made to anesthetized mice and peritoneal membrane was then incised. The cecum was gently externalized for optimal localization of the colon. Sterile foil with a narrow opening in the middle was placed over the trunk of the mouse so that the opening became in tight contact with a segment of the proximal colon.

A Silver LED (Prizmatix) was used as a 415 nm light source. Proximal colon was exposed to light for 3 min while kept wet with sterile NaCl solution. Care was taken not to illuminate other parts of the intestine or the lymph nodes in mesenterium. The abdominal cavity and the skin were closed with absorbable suture (Vicryl™, Ethicon) in two layers. Buprenorphine (0,3 mg/ml) was administered twice daily after the operation. Tissues were collected 24 h later.

### In situ hybridization for identification of bacterial DNA in lymph nodes

Tissues were collected into formalin, fixed for 24 h and then moved to 70% EtOH before casting into paraffin blocks. Sections were cut onto silane-coated glasses and deparaffinated. Alexa Fluor 647-labeled DNA probe reacting with a region of 16S-RNA gene common to all eubacteria (EUB-338: 5′-GCTGCCTCCCGTAGGAGT-3′) was applied onto the sections in hybridization buffer (20 mM Tris-HCl, 0.9 M NaCl, 0.1% SDS, 20% formamid, pH 7.4). Alexa Fluor 555-labeled scramble probe (5′-AGCCGTGTTGCCGTAGCC-3′) was used as a control (Supplementary Fig. 3). Sections were topped with a cover glass and incubated at 50 °C o/n. Cover glass was then removed and sections were washed once with wash buffer (20 mM Tris-HCl) and 3 times with PBS. Stained sections were mounted with ProLong Diamond Antifade Mountaint with DAPI (Molecular Probes, USA) and visualized with Nikon Eclipse Ti-2 microscope (Japan) and photographed with Hamamatsu sCMOS Orca-Flash4.0 camera (Japan).

### Identification of *C. rodentium*-derived fluorescence in pancreatic lymph nodes and myeloid cell populations

To study the translocation of bacterial components from the gut to draining lymph nodes, GFP plasmid (pAIDkiGFP4) was transfected to *C. rodentium* by heat shock. GFP-expressing *C. rodentium* was administered to recipient mice orally similarly to other experiments. Lymph nodes were collected 6 days after infection, and frozen lymph node sections (8 µm) were stained with APC conjugated F4/80 antibody to co-localize GFP-expressing bacteria and macrophages. Stained sections were imaged as described above. Dendritic cells were studied by flow cytometry as described below.

### Activation of adoptive transferred BDC2.5 cells

Lymph nodes were harvested from NOD.BDC2.5 mice, and a single cell suspension was prepared. Lymph node cells were stained with CellTrace™ CFSE Cell Proliferation Kit (Thermo Scientific, USA) according to manufacturer’s instructions. 1 × 10^6^ viable cells were injected into the tail vein of recipient NOD mice. Mice were treated daily with 20 µg FTY720 i.p. (Cayman Chemical Company, USA) to prevent lymphocyte egress from LNs. The percentage of BDC2.5 cells proliferating in indicated lymph nodes was analyzed 5 days after adoptive cell transfer.

### Flow Cytometry

Cells were stained with mixtures of fluorescently labeled monoclonal antibodies (Abs) and analyzed by flow cytometry. Shortly, single-cell suspension was prepared from the LN by pressing tissue through a metal mesh after collagenase digestion (100 µg/ml, 10 min, 37 °C, Sigma-Aldrich, USA). Surface receptors were stained with selected antibodies (see Supplementary Table 1.) and Zombie Red live/dead dye (Biolegend, USA) for 15 min at 4 °C in the dark. For IFNγ staining, cells were stimulated for 4 h (37 °C) in DMEM 10% FCS, and Cell Activation mixture (Cat#423304; BioLegend). Intracellular staining was done using Transcription factor buffer set (BD Biosciences, USA) according to manufacturer’s instructions. Stained cells were analyzed by flow cytometry (LSR Fortessa, BD Biosciences, USA; NovoCyte, Acea, USA) and analyzed with FlowJo (FlowJo LLC, USA) and NovoExpress (Acea, USA). For gating strategy of DC and macrophages and intracellular staining of T-cells for IFNγ, see Supplementary Figs. 4, 5.

### RNA isolation and quantitative PCR

Cytokine expression in pancreatic lymph nodes (PaLN) was analyzed with quantitative PCR. PaLNs from control or *C. rodentium*–infected mice were collected 7 days after gavage into RNA later™ (Qiagen, USA). RNA was isolated by PowerLyzer® Tissue & Cells RNA Isolation Kit (MoBio, USA) and genomic DNA was removed with DNase Max Kit (Qiagen, USA) according to manufacturer’s instructions. RNA was used for First Strand cDNA synthesis in a reaction applying Maxima Reverse Transcriptase and oligo-dT primers (Thermo Fisher, USA). cDNA and LightCycler®480 SYBR Green I Master (Roche, Switzerland) solution was used for qPCR, and the amplified product was detected using LightCycler®480 (Roche, Switzerland). Primer details are given in supplementary table 2. Ct values were normalized to β-actin expression and relative expression of target gene was calculated using 2^-ΔΔCt^ method.

### Ethical considerations

Pre-analgesia, sterile techniques, and inhalation anesthesia were applied for surgical operations. All procedures were approved by the National Project Authorization Board of Finland (License: ESAVI/19866/2019) in accordance with the EU Directive 2010/63/EU on the protection of animals used for scientific purposes.

## Supplementary information

Supplementary information
