# Supplementary material for: Infection with the enteric pathogen C. rodentium promotes islet-specific autoimmunity by activating a lymphatic route from the gut to pancreatic lymph node
Source: Mucosal Immunol. 2022 Feb 9;15(3):471–9. doi: 10.1038/s41385-022-00490-2 (PMC9038524; doi:10.1038/s41385-022-00490-2)
Supplement: Supplementary file 1 — Supplementary information [file 41385_2022_490_MOESM1_ESM.pdf]

Supplementary information

Supplementary Table 1 Monoclonal antibodies used in flow cytometry

Specificity	fluorophore	clone	Company	Experiment
**CD45**	Brilliant violet 785	30-F11	BioLegend	BDC2.5 transfer, DC activation, DC activation, GFP analysis, KikGR, OVA, OT-I transfer, T cell activation
**TCRβ**	AlexaFluor 700	H57-597	BioLegend	BDC2.5 transfer, T cell activation
**TCRβ**	Brillian violet 510	H57-597	BioLegend	GFP analysis, OVA
**CD3**	PE-Dazzle594	17A2	BioLegend	DC activation, KikGR
**CD3**	BV421	17A2	BioLegend	OT-I transfer
**CD4**	AlexaFluor 488	GK1.5	BioLegend	T cell activation
**CD4**	PE-Cy5	GK1.5	BioLegend	BDC2.5 transfer, OT-I transfer
**CD8**	APC	53-6.7	BioLegend	OT-I transfer, T cell activation
**CD11b**	AlexaFluor 700	M1/70	BioLegend	KikGR
**CD11b**	FITC	M1/70	BioLegend	OVA
**CD11c**	APC-Fire750	N418	BioLegend	GFP analysis, KikGR,
**CD11c**	Brilliant violet 605	N418	BioLegend	DC activation, OVA
**CD19**	PE-Dazzle594	6D5	BioLegend	BDC2.5 transfer, DC activation, KikGR, T cell activation
**CD19**	Brillian violet 510	6D5	BioLegend	GFP analysis, OVA
**CD64**	PE-Cy7	X54-5/7.1	BioLegend	GFP analysis, KikGR, OVA
**CD80**	APC	16-10A1	BioLegend	DC activation
**CD86**	APC-Fire750	GL-1	BioLegend	DC activation
**CD103**	Brilliant violet 421	2E7	BioLegend	OVA
**CD103**	Brilliant violet 510	2E7	BioLegend	KikGR
**CD103**	APC	2E7	BioLegend	GFP analysis
**MHCII***	PE	OX-6	BioLegend	DC activation, GFP analysis, OVA,
**MHCII**	Brilliant violet 421	M5/114.15.2	BioLegend	KikGR
**XCR1**	Brilliant violet 650	ZET	BioLegend	KikGR, OVA
**XCR1**	Brillian violet 421	ZET	BioLegend	GFP analysis
**IFNγ**	PE	XMG1.2	BioLegend	T cell activation

* anti-Rat RT1B, this antibody reacts with NOD mouse MHCII haplotype I-Ag7

Supplementary Table 2. Primers used for quantitative PCR

Gene	Forward 5’ → 3’	Reverse 5’ → 3’
Il1b	CAG GCA GGC AGT ATC ACT CA	GGGT GCT CAT GTC CTC ATC CT
Il10	GG CCA CAG TTT TCA GGG AT	AGG GCC CTT TGC TAT GGT GT
Il12	AAC TTG AGG GAG AAG TAG GAA TGG	GGA AGC ACG GCA GAA TA
Il18	CTG GGG TTC ACT GGC ACT T	TGG AGA CCT GGA ATC AGA CA
b-act	CTA AGG CCA ACC GTG AAA AG	ACC AGA GGC ATA CAG GGA CA

Supplementary Figure 1. Injection of FITC-Dx into the wall of small intestine loads into siMLN and not in PaLN. FITC-Dx was injected subserosally into 6 locations to cover the whole small intestine. (A) FITC-Dx accumulates under subcapsular sinus of the siMLN and lymphatic vasculature from the small intestine. (B) No FITC-Dx can be seen in PaLN (PaLN were marked with black ink and appear black). In B, orientation of the tissue is changed for better visualization of the PaLN. Yellow dotted line represents pancreas and green dotted line PaLN.

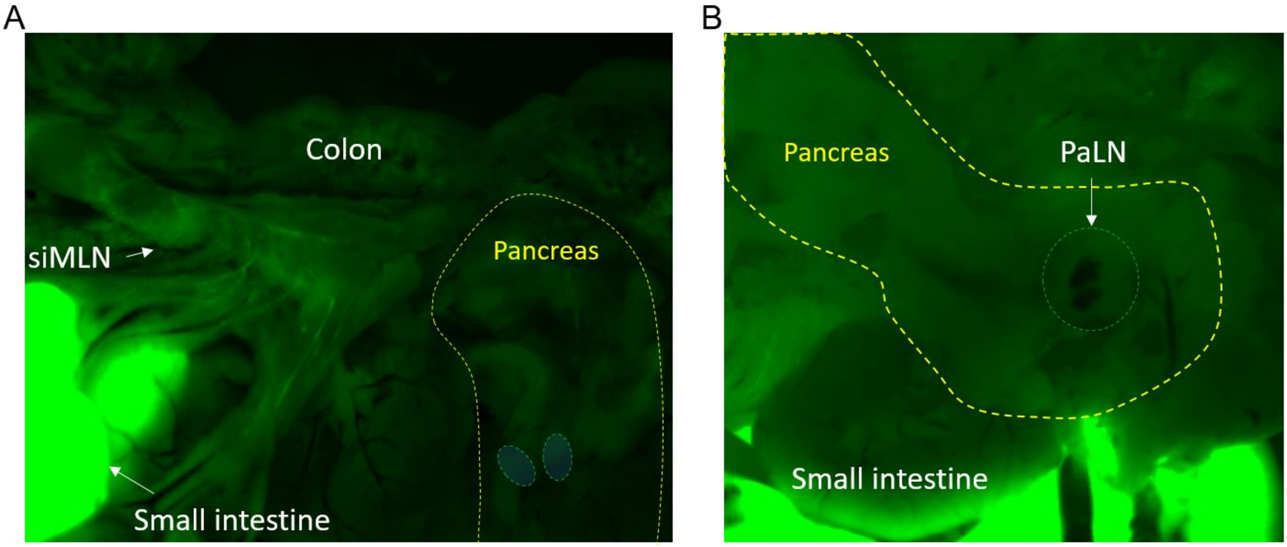

Supplementary Figure 2. Effects of *C. rodentium* on gut microbiota composition in NOD mice show alterations in Phyla Verrucomicrobia and Proteobacteria and in several other bacterial genera. Gut microbiota was analysed 14 days after C. rodentium administration using 16S-RNA parallel sequencing. Briefly, DNA was isolated from stool samples using QIAamp DNA Stool Mini Kit (Qiagen, Germantown, MD, USA) and after amplification of V3 and V4 regions 16S-RNA parallel sequencing was performed on an Illumina MiSeq system (Illumina, San Diego, CA, USA) as described previously [1].

(A) Uncensored pie chart analysis of gut microbiota on phylum level. The relative abundance of 6 most abundant phyla and “others” (left), and their mean percentages with p-values (right). (B) Genus-level identifications in phylum Verrucomicrobia show *Akkermansia* as the only identified genus. (C) The presence of *Citrobacter* is seen only in mice inoculated with it. Data are derived from genus-level identifications in phylum. (D, E) Genus-level expression levels which were identified with a statistically significant difference in abundant (D) and in less abundant (E) genera.

Contents of transverse colon were removed 14 days after mice were infected orally with *C. rodentium*. Microbiota was profiled by 16SRNA gene parallel sequencing (see Materials and methods).

A

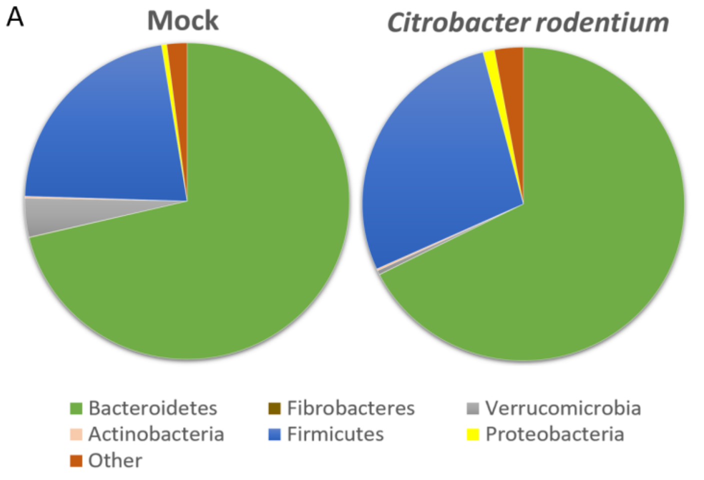

Phyla	Mock	*C. rodentium*	p-value
Acidobacteria	0,035	0,024	0,224
Bacteroidetes	71,287	67,547	0,427
Fibrobacteres	0,054	0,028	0,050
Ignavibacteriae	0,002	0,990	0,330
Verrucomicrobia	3,971	0,423	0,001
Actinobacteria	0,185	0,197	0,654
Aquificae	0,045	0,039	0,467
Caldiserica	0,000	0,014	0,329
Chloroflexi	0,073	0,058	0,055
Cyanobacteria	0,130	0,127	0,826
Deferribacteres	0,008	0,010	0,152
Firmicutes	21,976	27,768	0,246
Proteobacteria	0,522	1,160	0,005
Spirochaetes	0,050	0,057	0,484
Tenericutes	0,535	0,515	0,848

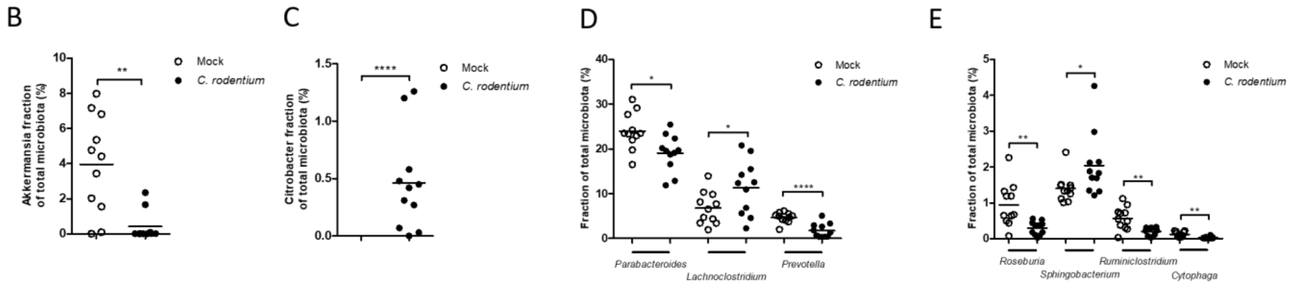

Supplementary Figure 3. *In situ* hybridization staining of lymph nodes and control. Red = EUB-338 probe for 16S-RNA encoding DNA (identifying bacterial DNA). Magenta = EUB-338 Scramble probe do not attach to bacterial DNA.

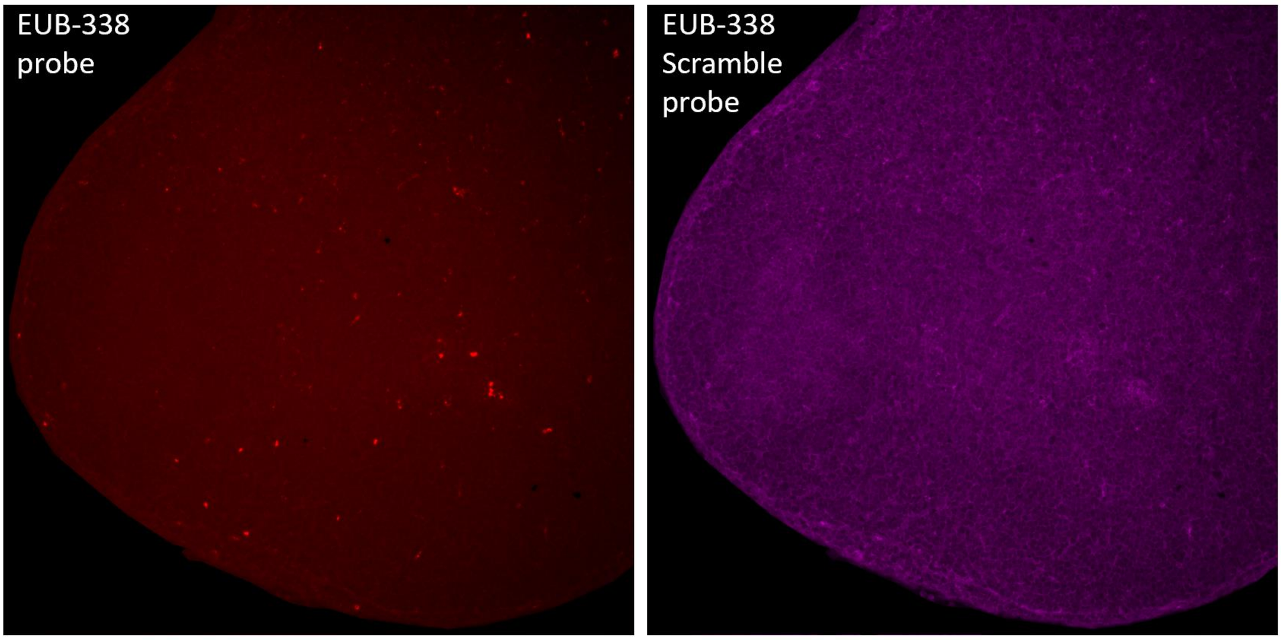

Supplementary Figure 4. Gating strategy for macrophages and DCs. Single cells were gated as CD45 positive cells and CD19-/CD3-/dead cells (zombie red) were excluded. Then MHCII positive cells were divided to CD64 positive macrophages and CD11c dendritic cells. CD11c DCs were phenotyped by their CD103 and XCR1 expression

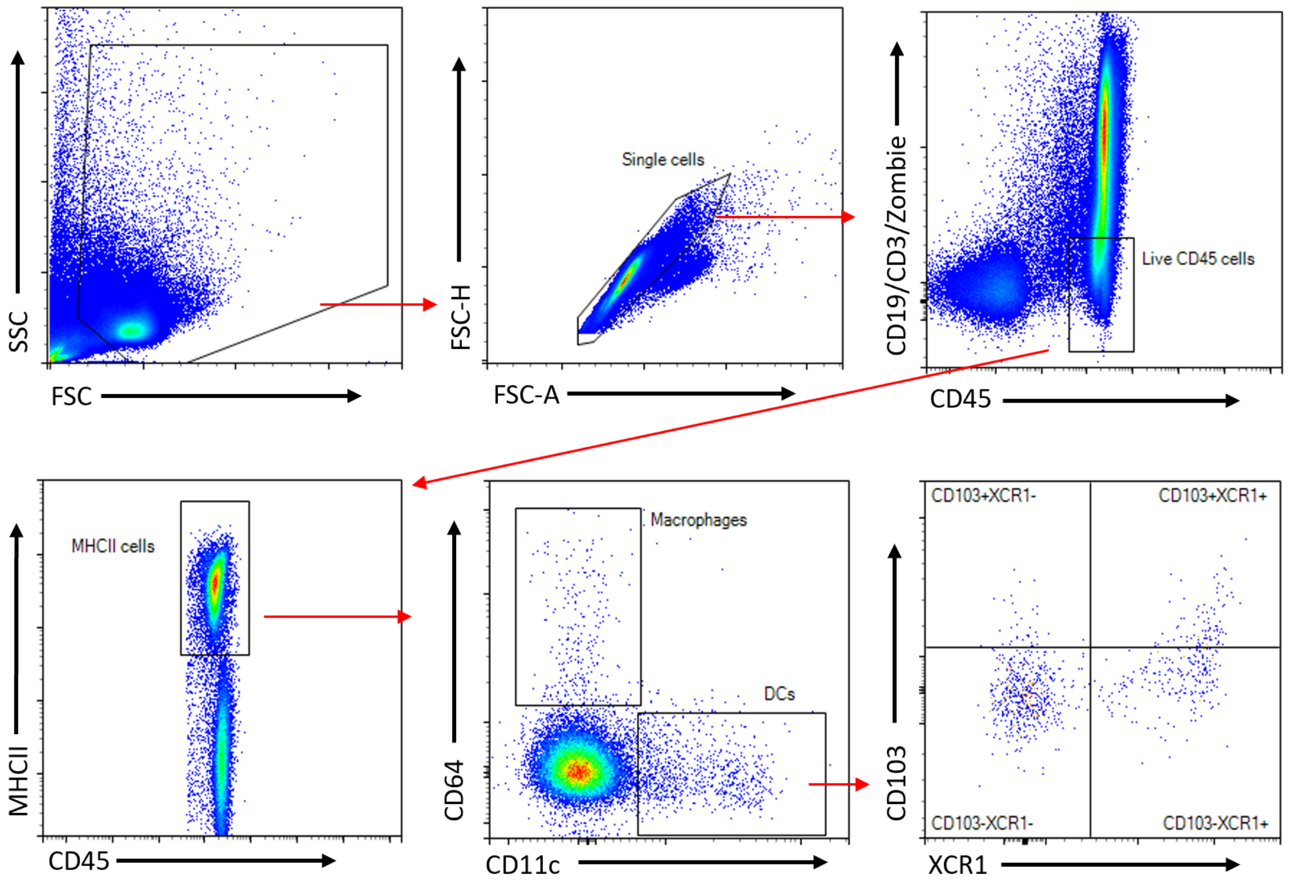

Supplementary Figure 5. Gating strategy for T cells and IFNγ staining. Single cells were gated as CD45 positive cells and CD19-/dead cells (zombie red) were excluded. Then TCRβ positive cells were divided to CD4 and CD8 cells to analyze IFNγ production separately in each cell population.

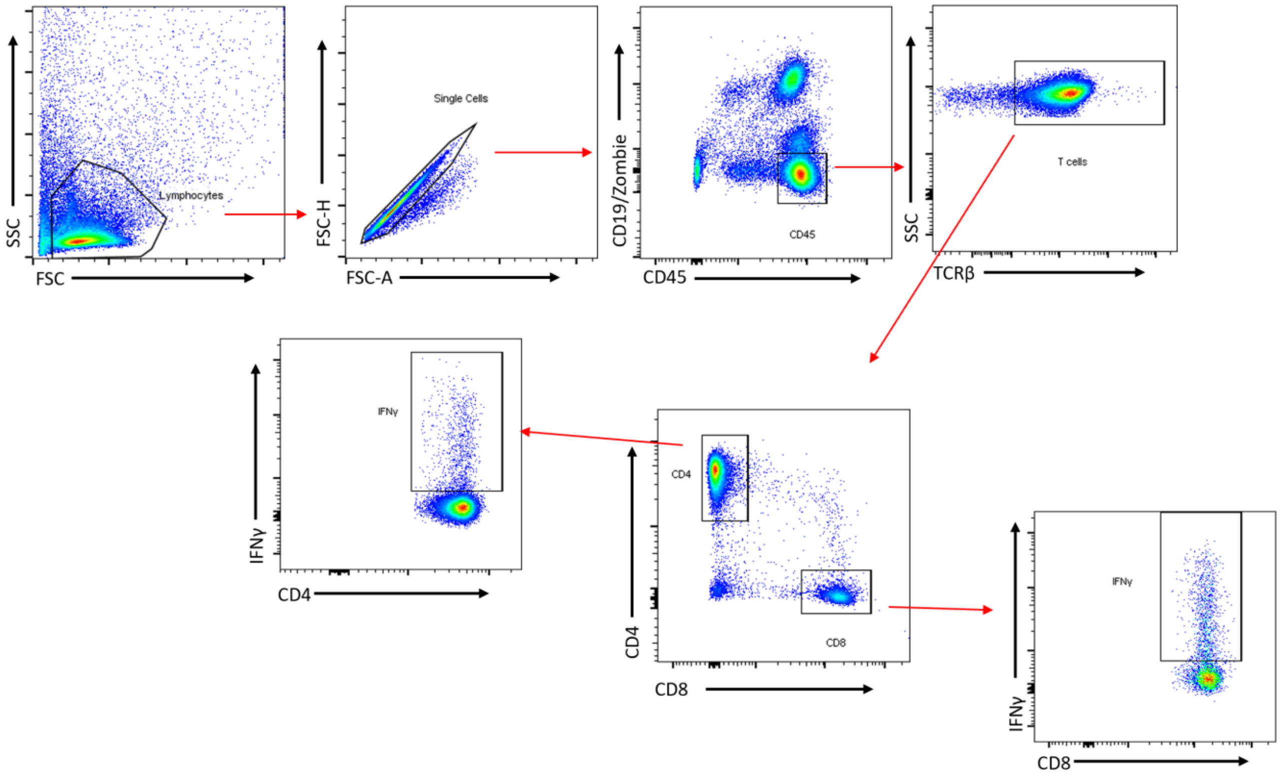

References

1. Toivonen, R.K. *et al.* Fermentable fibres condition colon microbiota and promote diabetogenesis in NOD mice. *Diabetologia* **57**, 2183-2192 (2014).
